# 3D assembly based on 2D structure of Cellulose Nanofibril/Graphene Oxide Hybrid Aerogel for Adsorptive Removal of Antibiotics in Water

**DOI:** 10.1038/srep45914

**Published:** 2017-04-03

**Authors:** Qiufang Yao, Bitao Fan, Ye Xiong, Chunde Jin, Qingfeng Sun, Chengmin Sheng

**Affiliations:** 1 School of Engineering, Zhejiang A & F University, Hangzhou, Zhejiang Province, 311300, P. R. China; 2 Key Laboratory of Wood Science and Technology, Zhejiang Province, 311300, P. R. China

## Abstract

Cellulose nanofibril/graphene oxide hybrid (CNF/GO) aerogel was fabricated via a one-step ultrasonication method for adsorptive removal of 21 kinds of antibiotics in water. The as-prepared CNF/GO aerogel possesses interconnected 3D network microstructure, in which GO nanosheets with 2D structure were intimately grown along CNF through hydrogen bonds. The aerogel exhibited superior adsorption capacity toward the antibiotics. The removal percentages (R%) of the antibiotics were more than 69% and the sequence of six categories antibiotics according to the adsorption efficiency was as follows: Tetracyclines > Quinolones > Sulfonamides > Chloramphenicols > β-Lactams > Macrolides. The adsorption mechanism was proposed to be electrostatic attraction, p-π interaction, π-π interaction and hydrogen bonds. In detail, the adsorption capacities of CNF/GO aerogel were 418.7 mg·g^−1^ for chloramphenicol, 291.8 mg·g^−1^ for macrolides, 128.3 mg·g^−1^ for quinolones, 230.7 mg·g^−1^ for β-Lactams, 227.3 mg·g^**−**1^ for sulfonamides, and 454.6 mg·g^−1^ for tetracyclines calculated by the Langmuir isotherm models. Furthermore, the regenerated aerogels still could be repeatedly used after ten cycles without obvious degradation of adsorption performance.

The global occurrence in water resources of antibiotics, has raised concerns about potentially negative effects on aquatic ecosystems including fish egg production inhibition and sex reversal of males as well as human health^1^,^2^,^3^,^4^,^5^. Therefore, it is urgent to put forward some rational and feasible suggestions for antibiotic pollution control. Recently, antibiotic is decontaminated by a variety of methods such as adsorption^6^, catalytic degradation^7^, photo-catalytic degradation^8^, advanced oxidation^9^,^10^, and biodegradation^11^. Among these various methods, adsorption is a superior and widely used method owing to its accessibility, environmentally benign and high efficiency. In fact, a number of (chemical or physical) sorbent materials, including activated carbons^12^,^13^, multiwalled carbon nanotubes^14^,^15^, activated sludge^16^, bamboo charcoal^17^, and graphene oxide^18^, have been applied for eliminating antibiotics from aqueous solutions. However, suffered from low removal capacities, difficult separation, the secondary environmental pollution and unsatisfactory recycling ability, it has been greatly hampered for these adsorbents in practical applications. Consequently, it is an urgent demand to develop new adsorbents with reusability and efficiency to remove antibiotics in water.

Aerogels, porous nanostructured materials, have been employed in many applications due to their superior properties like low density, high specific surface area, and excellent adsorption^19^. With high fragility and tendency to collapse easily, inorganic aerogels produced from silica^20^ and metal oxides^21^ are limited their use in applications. To order to achieve high flexibility and low density, several studies are attempting to prepare organic aerogels from biomaterials such as cellulose fibers. Hence, aerogels based on cellulose nanofibers (CNF) networks are recently developed. As the CNF aerogel has outstanding physical properties, including high aspect ratio, high surface-to-volume area, it shows an attractive flexibility, ductility, hierarchically porous structure, and many other excellent features^22^. As a result, materials based on CNF aerogels with 3D structures are the current focus of a tremendous surge of interest in adsorption applications.

With a single atomic layer of sp^2^-hybridized carbon arranged in a honeycomb structure, graphene oxide (GO) nanosheets with unusual properties such as excellent and mechanical properties have been applied to a lot of fields^23^,^24^,^25^. A lot of the researches in the last few years have proved that GO could be a promising material to adsorb pollutants from water, due to its extreme hydrophilicity, ultrahigh theoretical surface area and abundant surface oxygen-containing groups^26^,^27^. In previous study, GO-based materials were used as an efficient adsorbent for antibiotics (tetracyclines)^19^, dyes (methylene blue, Basic Red 12, and triphenylmethane)^28^,^29^ and heavy metal ions (copper, zinc, cadmium and lead)^30^. However, there is a little researches of its potential application for removal of multiple antibiotics in water.

In this work, CNF/GO was prepared by using one-step ultrasonication method, which showed a 3D porous networks structure. The results demonstrated the scientific significance of the adsorption properties within the CNF/GO aerogel for application in removal of antibiotics. In addition, the behavior of GO/CNF aerogel toward removal antibiotics was estimated. Moreover, the GO/CNF could be regenerated several times using an alkali washing procedure with little loss in multiple antibiotics removal performance.

## Results and Discussion

**Figure 1:**
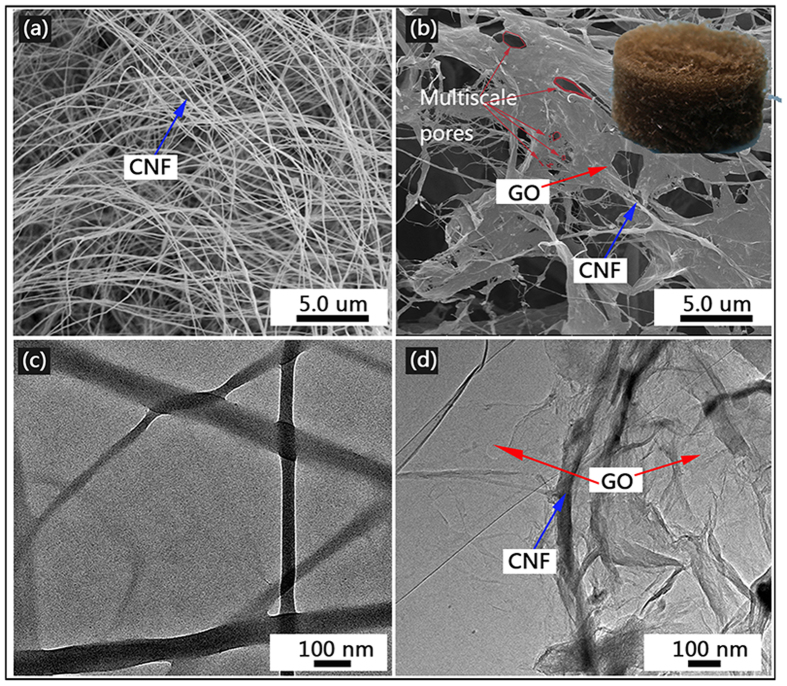
SEM images (**a**) and TEM (**c**) images of the CNF aerogel and SEM images (**b**) and TEM (**d**) images of the CNF/GO aerogel, respectively.

Figure 1 shows SEM images (a and b) and TEM images (c and d) of the aerogels, respectively. In Fig. 1a, the CNF presents a 3D network structure, in which individual flexible fibrils have an mean aspect ratio of roughly 1657 (58 μm in length and 35 nm in diameter). For CNF/GO aerogel, there is an interconnected 3D porous networks microstructure with pore sizes ranging from nano- to macro-scale, in which GO sheets are supported by CNF to form a porous network sheets (Fig. 1b). Moreover, the width of GO sheets is roughly 4.5 μm, which its color is grey and the density is 1.84 mg/cm^3^ (the inset in Fig. 1b). Further learned from TEM images of the aerogels and, CNF/GO aerogel shows that CNF are covered by GO nanosheets to form an interconnected planar ultrathin network sheets (Fig. 1d) by comparing to CNF of porous networks structure (Fig. 1c).

**Figure 2:**
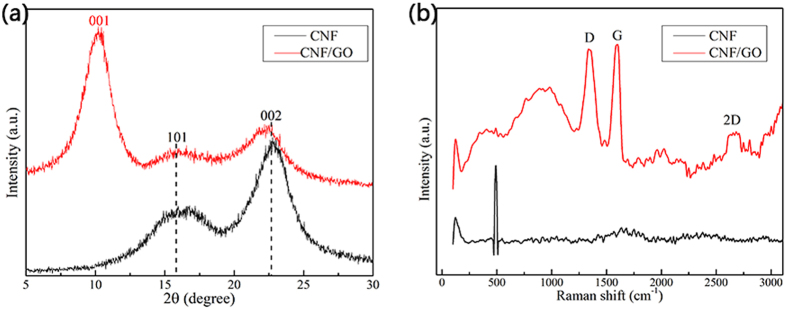
XRD patterns (**a**) and Raman spectra (**b**) of CNF and CNF/GO aerogel.

XRD patterns (a) and Raman spectra (b) of the CNF and the CNF/GO aerogel are shown in Fig. 2. In Fig. 2a, the diffraction peaks at about 22° and 16° are attributed to the typical reflection planes (002) and (101) of cellulose I based on the JCPDS data (03-0289)^31^. Just for CNF/GO aerogel, an intense peak at 2θ = 10.3° could be observed, corresponding to the (001) diffraction planes of GO^32^. There is no obvious peaks of cellulose in Raman spectrum of the CNF, which could be because of used laser wavelength. Compared to the CNF, the Raman spectrum of the CNF/GO aerogel (Fig. 2b) shows three new peaks. The G band at approximately 1600 cm^−1^ originated from the in-plane vibration of sp^2^ carbon atoms and is a doubly degenerate phonon mode (E_2g_ symmetry) and simultaneously the presence of isolated double bonds. The 2D band (at roughly 2700 cm^−1^) originates from a two phonon double resonance Raman process and the D peak at 1355 cm^−1^ is the symmetry A_1g_ mode of the sp^3^ carbon atoms involving phonons near the K zone boundary^33^.

**Figure 3:**
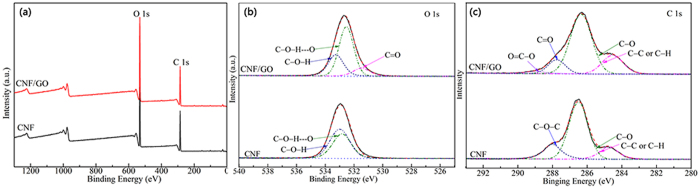
(**a**) Survey scan, (**b**) O1s and (**c**) C1s XPS spectra of CNF and the CNF/GO aerogel.

X-ray photoelectron spectroscopy (XPS) is used for characterizing the surface chemistry of CNF/GO aerogel. The fully scanned spectra in Fig. 3a illustrates that both the O and C elements existed simultaneously of the CNF and the CNF/GO aerogel. The variation of C/O ratio reveals the changes in the elemental composition of the specimens. Compared with the C/O ratio of 1.33 in the CNF, the C/O ratios for the CNF/GO is 1.5, further indicating coexist of CNF and GO in CNF/GO during the one-step ultrasonic treatment. The high resolution XPS spectra of O1s in the CNF and the CNF/GO are shown in Fig. 3b. Obviously, oxygen atoms in CNF are linking to carbon atoms, but because some of the oxygen atoms of the hydroxyl groups involve in the formation of hydrogen bonds OH ... O, while others simply exist in a free hydroxyl group. Therefore, XPS Energy spectrum of O1s could be fitted considering the following contributions: C–OH...O (532.8 eV) and C–OH (533.0 eV). Compared to CNF, The C–OH...O peak intensity of CNF/GO has been increased obviously, which indicates that GO are effectively combined with CNF through hydrogen bonds. In addition, there is a new peak of at 531.4 eV, which could be assigned to O=C derived from GO in CNF/GO.

The high-resolution XPS C1s spectra of CNF and CNF/GO aerogel are provided in Fig. 3c. The curves are fitted considering the following contributions: C=C or C–C or C–H (284.4 eV), C–O (286.5 eV), C=O (287.8 eV), O–C=O (289.8 eV). The C=C/C–C peak intensity of CNF/GO increased obviously compared with CNF, which indicates that GO are effectively combined with CNF. In addition, there is still a large number of oxygen containing functional groups, which mainly derive from CNF in the CNF/GO. The other peaks at 287.8 and 289.1 eV are attributed to C=O bonds and O-C=O bonds, respectively, which are typical functional groups in GO. In conclusion, GO are intimately combined with CNF through hydrogen bonds.

**Figure 4:**
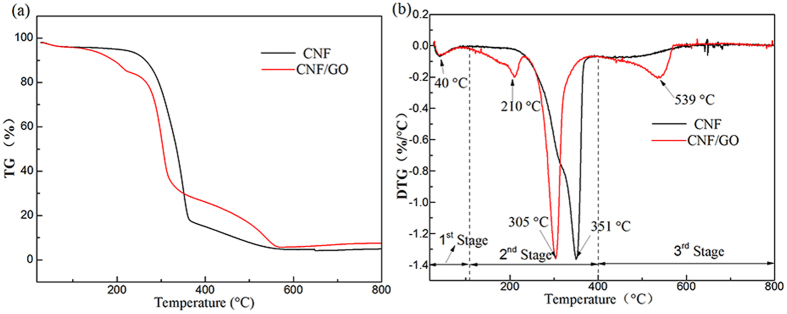
TG (**a**) and DTG (**b**) curves of CNF and CNF/GO aerogel.

Figure 4 shows the thermogravimetric (a) and differential thermogravimetric analysis (b) (TG–DTG) curves of CNF and CNF/GO aerogel. In the first stage (25 °C to 110 °C), little weight loss (4%) in both specimens are observed which are attributed to the evaporation of moisture in the surfaces of the specimens. In the second stage (110 °C to 400 °C), owing to pyrolysis of CNF, main weight loss has happened and the maximum pyrolysis rate occurred at 351 °C and 305 °C with weight loss reaching 80.9% for CNF and 69.8% for CNF/GO aerogel, respectively (Fig. 4b). Simultaneously, the smaller pyrolysis rate occurs at 210 °C with weight loss reaching 8.5% for the CNF/GO, which belongs to the loss of oxygen-containing groups from the GO^34^. In the third stage (400 °C to 800 °C), the mass percentage of pyrolysis residue are about 4.9 and 7.5 mass% for CNF and CNF/GO, respectively. These results lead to the conclusion that the GO are successfully combined with CNF.

**Figure 5:**
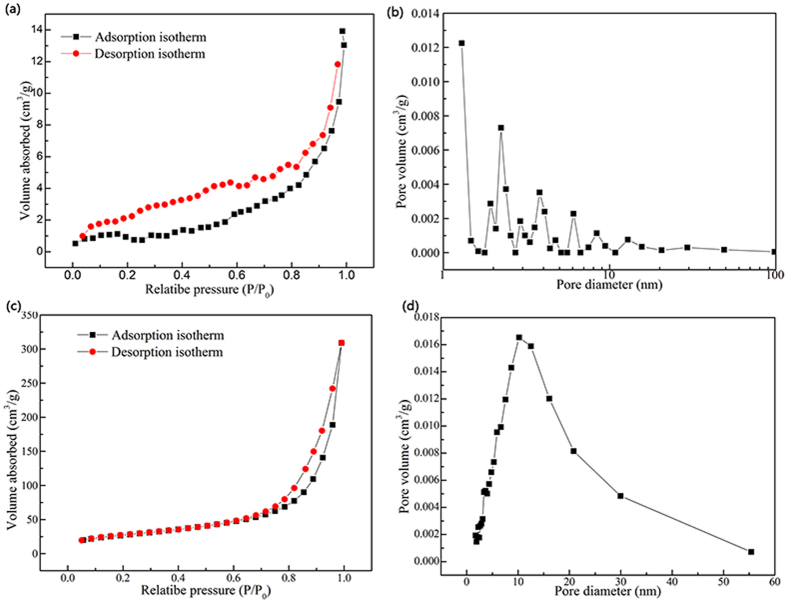
Nitrogen adsorption–desorption isotherms and BJH (Barret–Joyner–Halenda) desorption pore size distribution of CNF (**a** and **b**) and CNF/GO aerogel (**c** and **d**), respectively.

Figure 5 presents the N_2_ adsorption–desorption isotherms (a and c) and pore diameter distributions (b and d) of the aerogels. The adsorption isotherms both of CNF (Fig. 5a) and GO/CNF aerogel (Fig. 5c) are attributed to type-IV adsorption isotherm, according to the IUPAC classification. Compared with the CNF, the CNF/GO aerogel show larger the S_*BET*_ and pore volume. For instance, the S_*BET*_ and pore volume of the CNF are as low as 3.3 m^2^·g^−1^ and 0.03 cm^3^·g^−1^, respectively. For CNF/GO aerogel, the S_*BET*_ increased to 97.5 m^2^·g^−1^ and the values of pore volume are as high as 0.4 cm^3^·g^−1^. Simultaneously, For CNF, the pore size distribution shows that there are multi-scale pores located in the range (1.6–100 nm) shown in Fig. 5b, indicating that there are consisted of micropores, mesopores and micropores in the CNF. For CNF/GO aerogel, the pore size distribution (Fig. 5d) is mainly lied in the mesoporous range, in which a notable single peak appears at 10.2 nm. Obviously, for CNF/GO instead of CNF, there are not only more mesopores, but also more uniform pore structure. These results indicates CNF/GO aerogel with ultrahigh surface areas might be effective in adsorbing antibiotics.

**Figure 6:**
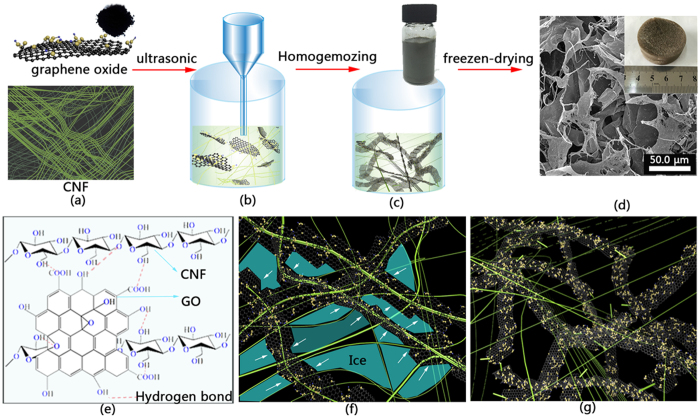
Schematic showing the synthetic steps of the CNF/GO aerogel: (**a**–**d**); (**e**) CNF combined with GO through hydrogel bond; (**f**) schematic formation of the 3D structure; (**g**) 3D structure of CNF/GO.

Based on the above characterization results, the fabrication process of the CNF/GO aerogel is shown schematically in Fig. 6. It has been shown that CNF could help to disperse GO with sonication treatment, creating a uniform CNF/GO dispersion (Fig. 6a,b and c). During the ultrasonic treatment, the energy of ultrasound is transferred to the CNF chains and GO through cavitation effect with approximately 10–100 kJ/mol, which is within the hydrogen bonds energy scale^35^. As a result, the abundant oxygen containing groups in GO could interact with the hydroxyl groups in cellulose through hydrogen bonds (Fig. 6e). Afterwards, the CNF/GO aerogel is obtained by freeze-drying a CNF/GO dispersion (Fig. 6d). When an aqueous suspension of CNF/GO is frozen, phase separation could result in the rejection of solid CNF/GO from the forming ice, which are then accumulated between the growing ice crystals (Fig. 6f). Moreover, the margin of CNF/GO is higher than the percolation threshold so that the entrapped CNF/GO could form interconnected 3D network microstructure oriented along the freezing direction^36^. Subsequent sublimation of ice resulted in the formation of a porous solid material (Fig. 6g). Consequently, the prepared CNF/GO aerogel possesses interconnected 3D network microstructure.

**Figure 7:**
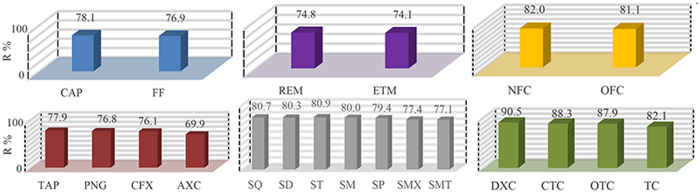
The R% of the antibiotics by the CNF/GO aerogel.

Figure 7 shows the removal percentage (R%) of antibiotics by the CNF/GO aerogel. 21 kinds of antibiotics could in different degree be absorbed by the CNF/GO aerogel. The R% of these antibiotics ranges from 69–90.5%, assigning to that of AXC and DXC, respectively. The sequence of six categories antibiotics according to the adsorption efficiency is as follows: Tetracyclines > Quinolones > Sulfonamides > Chloramphenicols > β-Lactams > Macrolides, which are possible attributed to different conjugated structure and different amounts of hydroxyl groups in six categories antibiotics. In details, for tetracyclines, the sequence of adsorption efficiency is as follows: DXC (90.5%) > CTC (88.3%) > OTC (87.9%) > TC (82.1%), due to the different groups on the branched chain. For quinolones, the sequence is as follows: NFC (82.0%) > OFC (81.1%). For sulfonamides, the sequence is as follows: SQ (80.7%) > ST (80.9%) > SD (80.3%) > SM (80.0%) > SP (79.4%) > SMX (77.4%) > SMT (77.1%). For chloramphenicol, the sequence is as follows: CAP (78.1%) > FF (76.9%). For β-Lactams, the sequence is as follows: TAP (77.9%) > PNG (76.8%) > CFX (76.1%) > AXC (69.9%). For macrolides, the sequence is as follows: REM (74.8%) > ETM (74.1%). These results exhibit that more conjugated structures and hydroxyl groups of these antibiotics could be contributed to adsorption by CNF/GO aerogel. Obviously, the adsorption efficiency of tetracyclines is comparatively ideal.

**Figure 8:**
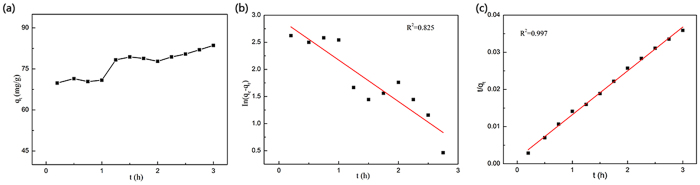
The adsorption kinetic curve (**a**) and the pseudo-first-order (**b**) and pseudo-second-order (**c**) kinetics models for adsorption of TC by CNF/GO aerogel.

TC is selected as a typical object to systematically study the adsorption kinetic and adsorption isotherm of CNF/GO aerogel for removing the antibiotics in water. Figure 8 shows the adsorption kinetics, the pseudo-first-order, and pseudo-second-order kinetics models for adsorption of TC by CNF/GO aerogel. Adsorption kinetic is shown in Fig. 8a and TC is gradually absorbed by CNF/GO until reaching to equilibrium concentration. The linear fitting results are plotted in Fig. 8b and c with the coefficients of determinations of 0.83 and 0.99, respectively. The obtained kinetics parameters of the pseudo-first-order kinetics model and pseudo-second-order kinetics model are listed in Table 1. Comparison of the correlation coefficients of the two kinetic models indicates that the pseudo-second-order kinetics model describes the data better than the first-order model, which relies on the assumption that adsorption is a chemical reaction, i.e. chemisorption. Therefore, quick adsorption of TC by the CNF/GO suggested chemical adsorption. The calculated adsorption capacity (q_*e*_) values estimated by the pseudo-second-order model is 84.8 mg·g^−1^. The rate constant of sorption (k) is 0.09 g·(mg·h)^−1^.

**Figure 9:**
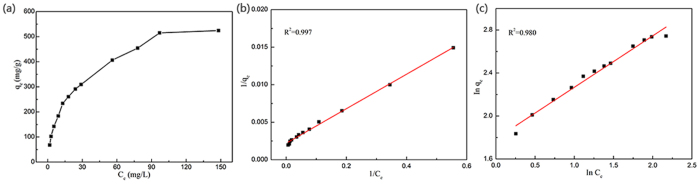
Adsorption isotherms of TC by CNF/GO aerogel (**a**) with corresponding fitted Langmuir (**b**), and Freundlich (**c**) models.

Adsorption isotherm shown in Fig. 9a, adsorption capacities increase with the incremental in equilibrium concentrations of adsorbates and the slopes of adsorption isotherms decreases gradually. Langmuir model is an ideal model, which possesses perfect adsorbent surface and monolayer molecule adsorption. As an empirical model, Freundlich model is used widely in the field of chemistry. The results of fitting these models are shown in Fig. 9, and the fitting parameters for TC are listed in Table 2. In the range of tested concentration, the Langmuir (R^2^ = 0.99) fitted the adsorption data well, while the Freundlich model (R^2^ = 0.98) fitted reasonably. Although the Langmuir model globally fits well, it does not give apparently a nice fit at high concentration levels, showing the limitation of the hypothesis about a monolayer adsorption. From Langmuir model, the ideal maximum adsorption capacity (q_*m*_) as a model fitting parameter is determined to be 454.6 mg·g^−1^. Using the Freundlich model, the obtained Freundlich constants (K_*F*_ is 61.4 L·mg^−1^ and n is 2.1) are higher than those reported by Ghadim^19^ (K_*F*_ is 21.4 L·mg^−1^ and n is 3.7).

**Figure 10:**
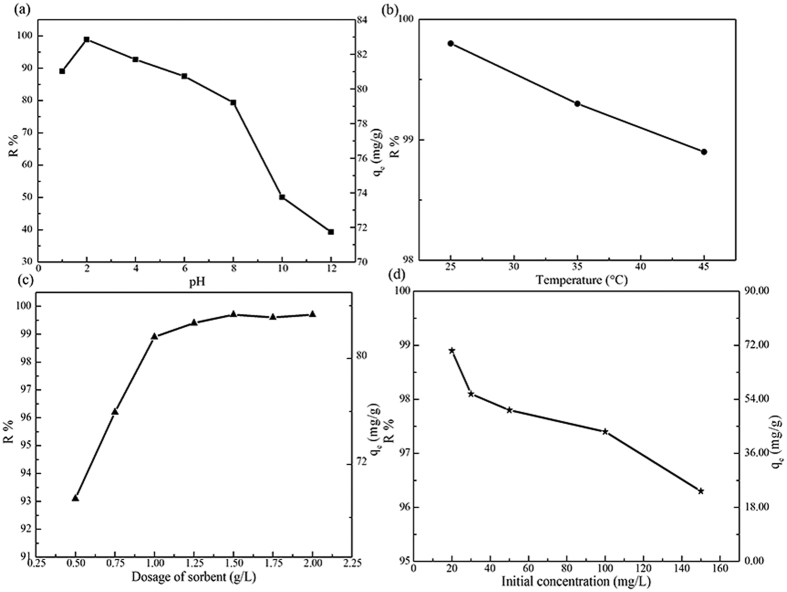
Effect of pH (**a**), temperature (**b**), and the effect of CNF/GO dose (**c**) and the initial concentration of TC (**d**) on antibiotic removal.

To study the effect of factors on antibiotic removal, TC is regarded as a behalf. In Fig. 10a, the TC adsorption is sensitive to the pH in the solution. The presence of H^+^ or OH^−^ ions in solution changes the surface charge of adsorbent. The highest R% for TC is seen with pH 2. With increasing pH, the R% gradually decreases. Under these conditions, the active sites on the absorbent surface changed to phonoxide (PhO^−^) and hydroxide (OH^−^) ions^37^.

Figure 10b shows the effect of temperature (25, 35 and 45 °C) on antibiotic removal. The adsorption capacity decreases with increasing temperature, which indicates that the adsorption is an exothermic process and the temperature at 25 °C favored the adsorption. This could be explained by considering that appropriate temperature may cause a swelling effect on the porosity and the pore volume of the adsorbent, which enables TC molecules to rapidly diffuse across the external boundary layer and within the internal pores of the CNF/GO aerogel. However, if temperature is such high that the microstructure of the adsorbent would be destroyed.

The effect of adsorbent dose on antibiotic removal is shown in Fig. 10c. The R% of TC is went up with increasing adsorbent dose. The positive correlation between adsorbent dose and R%, which could be due to an increase in the adsorbent surface area and availability of more adsorption sites. After a dose of 1 g/L, the R% is nearly constant. This is due to the non-availability of active sites on the adsorbent and establishment of equilibrium between TC molecules on the adsorbent and in the solution. As a result, the optimal adsorption is achieved when concentration of adsorbent is 1 g/L.

Figure 10d shows the effect of initial concentrations of TC on antibiotic removal. The R% of TC decreases with an increase in antibiotic concentration. This could be explained by considering that all adsorbents has a limited number of active sites and at certain concentration the active sites became saturated.

**Figure 11:**
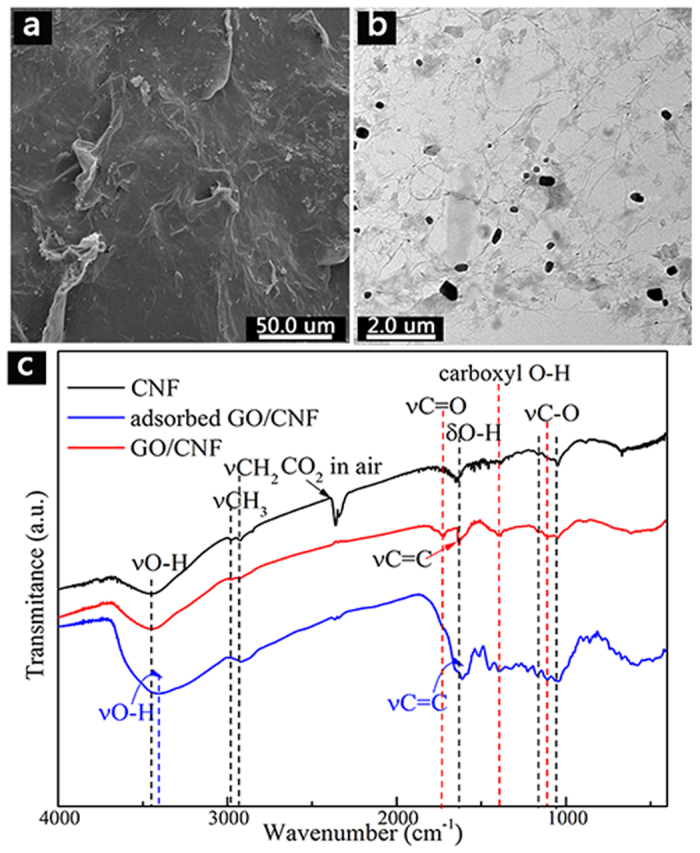
SEM image (**a**) and TEM image (**b**) of the adsorbed CNF/GO; the FTIR spectra of the CNF, the CNF/GO and the adsorbed CNF/GO (**c**).

To verify the adsorption mechanism, the absorbed CNF/GO is investigated by SEM, TEM and FTIR spectra (Fig. 11). Just for the microstructures, SEM image of the adsorbed CNF/GO shows the antibiotic scattered on the CNF/GO (Fig. 11a) as well as crimp of CNF/GO aerogel. Further, TEM image of the adsorbed CNF/GO exhibits the antibiotic is embedded in the network structure of CNF/GO (Fig. 11b). In the FTIR spectrum of CNF (Fig. 11c), the peaks at 2929 and 2895 cm^−1^ are assigned to C–H stretching vibrations. The peaks at 1155 and 1051 cm^−1^ are assigned to the vibrations of C–O. The broad band at 3442 cm^−1^ is associated with the hydroxyl groups and the peak at 1633 cm^−1^ is associated with tramolecular hydrogen bonds. The characteristic adsorption peaks in the spectrum of CNF/GO aerogel are similar to the curve of CNF apart from appearance of new bands at 1736 (C=O), 1639 cm^−1^ (C=C), and 1406 cm^−1^ (carboxyl O–H), suggesting that oxygen-containing groups of GO are introduced into the CNF. Moreover, the wider the broad peak at 3442 cm^−1^ in the spectrum of adsorbed NFC/GO (hydroxyl groups) could be assigned to the interaction between CNF and GO by hydrogen bonds.

**Figure 12:**
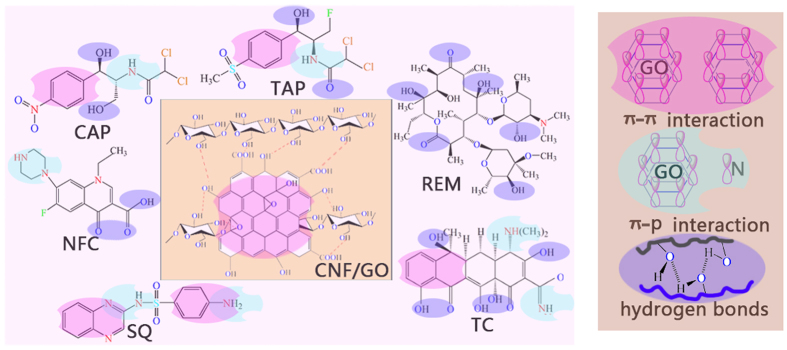
Schematic diagram of the CNF/GO aerogel for antibiotics adsorption.

After the adsorption of TC, the FTIR spectrum of the adsorbed CNF/GO exhibits many changes. The new adsorption peaks at 1460 cm^−1^ (C–N), 1624 cm^−1^ (C=O), and 3457 cm^−1^ (N–H) derives from TC, indicating that TC is absorbed by GO/CNF aerogel. Furthermore, the characteristic adsorption peak of GO at 612 cm^−1^ is ascribed to the aromatic rings ranging from 588 to 666 cm^−1^. It also could be seen that the adsorption band of the adsorbed CNF/GO at 1643 cm^−1^ (C=C) slightly increased, confirming that TC molecules containing C=C benzene rings with π electrons and N atom with lone pairs could be formed π-π stacking and p-π stacking with CNF/GO, in which there is the presence of π-π stacking. The molecule interactions between CNF/GO and TC discussed above during the adsorption process are schematically represented in Fig. 12.

The possible adsorption mechanism of the antibiotics by CNF/GO are based on the following properties. Firstly, the interconnected 3D networks structure of CNF/GO aerogel composed of micro-, meso-, and macropores, which facilitates the free diffusion of six categories antibiotics and guaranteed mass transport to the 3D cross-linking internal structure, and fully exposed active sites would enhanced the opportunity for antibiotics to contact with the macro-structure of CNF/GO via electrostatic attraction. Secondly, CNF/GO with primarily π–π stacking could act as electron acceptors and be advantageous for adsorbing the antibiotics with unsaturated double bond or conjugate structure (Fig. 12). In addition, a lots of hydroxyl groups both in CNF/GO aerogel afford formation of hydrogen bonds with antibiotics molecules. In brief, the CNF/GO aerogel for antibiotics adsorption are through electrostatic attraction, π–π stacking, π–p stacking and hydrogen bonds.

The maximum theoretical adsorption capacities (Q_*m*_) from Langmuir models as a model fitting parameter of the CNF/GO composite on 21 kinds of antibiotics are listed in Table 3. As could be seen, the Q_*m*_ range from 128.3–501.1 mg·g^−1^. Generally, the sequence according to the Q_*m*_ is as follows: Tetracyclines > Chloramphenicol > β-Lactams > Sulfonamides > Macrolides > Quinolones, which could be as result of the interaction between adsorbent and adsorbate. For example, the Q_*m*_ of TC by the CNF/GO composite is 454.5 mg·g^−1^, which is much higher than other adsorbents such as palygorskite^38^, kaolinite^39^, MCM-41^40^ and bamboo charcoal^18^. Such comparison suggests that CNF/GO might be an effective adsorbent for TC removal from contaminated water. However, this value is smaller than that by Smectite and Q_*m*_ of OTC is a little smaller than that by SWCNTs which illustrates the CNF/GO should be improved in the future research.

**Figure 13:**
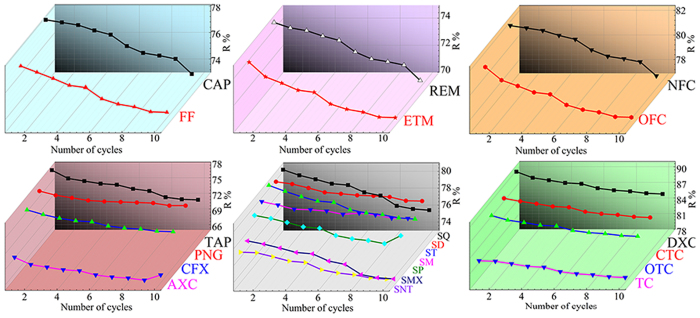
Reusability of CNF/GO for antibiotics removal in ten cycles.

Figure 13 presents the reuse potential of the CNF/GO studied on account of the importance of the recyclability, and economy for adsorbents via sequential cycles of adsorption – desorption. The R% of 21 kinds of antibiotics by CNF/GO during ten cycles are shown in Fig. 13. CNF/GO did exhibit a promising cycling behavior and lost 2–5.7% with mean of 3.8% in 10 cycles, which reduced by 2% for SM and 5.7% for TAP. In addition, the R% of TC by the recycled CNF/GO aerogel still remained at 78.9%, which only reduced by 3.9% compared to that in first cycle. The results indicates that the CNF/GO could be still efficiently reused through ten regeneration cycles, which illustrates the CNF/GO with excellent regeneration performance could be an efficient, economical, and potential adsorbent for antibiotics removal.

## Conclusion

In summary, cellulose nanofibril/graphene oxide hybrid (CNF/GO) aerogel has been prepared, which shows interconnected 3D network microstructure. The aerogel exhibits superior adsorption performance towards multiple antibiotics (six categories of antibiotics: Chloramphenicol, Macrolides, Quinolones, β-Lactams, Sulfonamides, and Tetracyclines) based on the integration of chemical reaction such as π–π stacking and hydrogen bonds as well as their interconnected 3D pore network microstructure. The maximum theoretical adsorption capacities (Q_*m*_) by the CNF/GO composite on theses antibiotics range from 128.3–501.1 mg·g^−1^. Especially, the Q_*m*_ of TC by the CNF/GO composite is 454.5 mg·g^−1^. Simultaneously, CNF/GO aerogel possesses reusability as well as ease of separation operation. Consequently, the CNF/GO aerogel could be an efficient, economical and potential adsorbent in antibiotics removal from wastewater.

## Materials and Methods

### Materials

21 kinds of antibiotics (six categories of antibiotics: Chloramphenicol, Macrolides, Quinolones, β-Lactams, Sulfonamides, and Tetracyclines) were shown in Table 4 and were supplied by Aladdin Industrial Co., Ltd. The moso bamboo (*Phyllostachys heterocycla*), obtained from Zhejiang Province in China, was used as the starting material to prepare cellulose nanofibrils (CNF). All other chemicals were analytical grade and used as received. De-ionized (DI) water was used in whole experiments. The 21 kinds of antibiotics (Table 4) were used in this study.

### Preparation of cellulose nanofibril/graphene oxide hybrid (CNF/GO) aerogel by one-step ultrasonication method

Cellulose nanofibril (CNF) and Graphene oxide (GO) were prepared according to the literature methodologies reported by W. Zhang^41^ and J. Chen^42^, respectively. To disperse the CNF and GO into DI water, in a typical process, 80 mg of CNF and 100 mg of GO were put in 100 mL DI water under vigorous ultrasonication for 30 min. In details, ultrasonic treatment was performed at 60 kHz with a 25-mm-diameter titanium horn under a duty cycle (i.e., a repeating cycle of 0.5 s ultrasonic treatment and 0.5 s shutdown) in an ice bath with an out-power of 1000 W. After ultrasonication treatment, a brown suspension of CNF/GO could be obtained. Afterwards, the brown suspension was transferred into several containers and then frozen at −30 °C for more than 5 h. Finally, the frozen specimens were freeze-dried using a lyophilizer (Scientz-10N, Ningbo Scientz Biotechnology Co., Ltd, China) at a condenser temperature of −55 °C. The specimens were kept frozen during the drying under a vacuum of 80 mTorr. The CNF/GO aerogel was obtained after freeze-drying for 48 h.

### Characterizations

The morphology of the aerogels were characterized by scanning electron microscopy (SEM, FEI, Quanta 200, USA) and transmission electron microscope (TEM, FEI, Tecnai G20, USA). The chemical functional groups were characterized by Fourier transform infrared spectroscopy (FTIR, Nicolet 5700, USA) measurements. Crystalline structures were identified by X-ray diffraction technique (XRD, Rigaku, D/MAX 2200, Japan) operating with Cu Kα radiation (λ = 1.5418 Å) at a scan rate (2θ) of 2° min^−1^ and the accelerating voltage of 40 kV and the applied current of 30 mA ranging from 10° to 30°. Raman spectra were recorded with a confocal Raman microscope (WiTec ALPHA 300) equipped with a piezo-scanner and 100× microscope objective (NA = 0.9) using 532 nm laser excitation. The surface elemental composition analyses were conducted based on the X-ray photoelectron spectroscopy (XPS, Thermo Fisher Scientific-K-Alpha 1063, UK) with an Al Ka monochromatic X-ray source, in which all of the binding energies were calibrated with reference to the C 1 s peak (284.8 eV). The thermal performances were examined using a thermal analyzer (TGA, SDT Q600, USA) under a nitrogen flow of 20 mL·min^−1^, and at heating-cooling rate of 10 °C/min ranging from 25 to 800 °C. The Brunauer–Emmet–Teller (BET) surface area (S_BET_) and pore properties of the aerogels were determined from N_2_ adsorption–desorption experiments at −196 °C using an accelerated surface area and porosimetry system (ASAP 2020, Micromeritics instrument Ltd., USA). All of the aerogels were outgassed at 90 °C for 10 h to remove any moisture or adsorbed contaminants prior to the surface area measurements. Meanwhile, the pore volume and pore-size distribution were estimated by the Barrett–Joyner–Halenda (BJH) method.

### The performance experiments

#### The adsorption experiment

All batch experiments of the antibiotics adsorption were carried out in 50 mL Erlenmeyer flask at a temperature controlled with water bath shaker at 160 rpm, each containing 8 mg CNF/GO aerogel and 40 mL of antibiotics solution with various concentrations (from 1 to 100 mg·L^−1^) and pH value, which could be adjusted to values ranging from 2 to 12 by 0.1 M NaOH or 0.1 M HCl with using a pH meter (PHSJ-4F, Shanghai, China). The mixed CNF/GO solutions were incubated with antibiotics overnight at 25 °C and covered by aluminum foil to refrain probable photo degradation of antibiotics. The final supernatant was separated from the solid phase and was regarded as the residual concentration of antibiotics and determined by HPLC (Aglient-1200, USA) and a UV–vis spectrophotometer (ERSEE TU-1900, China). The removal percentage (R%) and the amount of antibiotics absorbed (q_*e*_, mg·g^−1^) were calculated using Eqs (1) and (2), respectively:


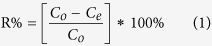



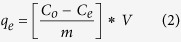


where C_*o*_ (mg·L^−1^) is the initial concentration of the antibiotics, C_*e*_ (mg·L^−1^) is the equilibrium concentration in solution of the antibiotics, V (L) is the volume of solution, and m (g) is the weight of the CNF/GO aerogel.

#### Adsorption kinetics experiments

Adsorption kinetics were conducted in triplicate. In order to investigate the adsorption process of antibiotic by CNF/GO aerogel, the pseudo-first-order, and pseudo-second-order kinetics models calculated by Eqs (3) and (4), respectively, and were represented as follows^43^,^44^:


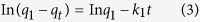



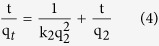


where k_*1*_ (h^−1^) and k_*2*_ (g·(mg·h)^−1^) are the rate constant of sorption in pseudo-first-order and pseudo-second-order model, respectively; q_*1*_ and q_*2*_ (mg·g^−1^) is the amount of antibiotic adsorbed at equilibrium, q_*t*_ (mg·g^−1^) is the amount of antibiotic adsorbed on the surface of the adsorbent at any time.

#### Adsorption isotherms experiments

At each adsorption cycle, the suspension was shaken under agitation speed of 160 rpm for 30 min at 25 °C. The supernatant was separated and analyzed by HPLC or UV-vis spectrophotometer, and replaced by fresh antibiotic solution. Subsequent adsorption cycles were performed until the CNF/GO aerogel reached its maximal adsorption capacity (exhausted aerogel). Adsorption experiments were conducted in triplicate. In a separate adsorption experiment, the exhausted aerogel suspension with the remaining antibiotic solution after the last adsorption cycle was used to test the recycle. The Langmuir and Freundlich models, two classic adsorption models, were used to describe the adsorption equilibrium. The mathematical representations of the Langmuir Eq. (5) and Freundlich Eq. (6) models^45^ were given below:


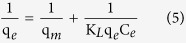



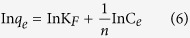


where q_*m*_ (mg·g^−1^) is the theoretical maximum adsorption capacity per unit weight of the adsorbent, K_*L*_ and K_*F*_ are adsorption constants of Langmuir and Freundlich models, respectively, and n is the Freundlich linearity index. Langmuir model is an ideal model, which possesses perfect adsorbent surface and monolayer molecule adsorption. As an empirical model, Freundlich model was used widely in the field of chemistry.

#### The effect of factors for the experiments

The effect of pH is as follows: C_antibiotics_: 20 mg/L, adsorbent: 4 mg, agitation speed; 160 rpm, agitation time: 24 h, T: 25 ± 2 °C, pH: 2 to 12. The effect of temperature is as follows: C_antibiotics_: 20 mg mg/L, adsorbent: 4 mg, agitation speed: 160 rpm, agitation time: 8 h, pH: 2, T: 25, 35, 45 °C. The effect of adsorbent dosage is as follows: C_antibiotics_: 20 mg/L, agitation speed: 160 rpm, agitation time: 24 h, T: 25 ± 2 °C, pH: 2, adsorbent: 0.25, 0.5, 1.0, 1.25, 1.5, 1.75, and 2 g/L. The effect of initial concentration of the TC is as follows: adsorbent: 4 mg, agitation speed: 160 rpm, agitation time: 24 h, T: 25 ± 2 °C, pH: 2, C_antibiotics_: 20, 30, 50, 100, and 160 mg/L.

### The recovery of the CNF/GO aerogel

The CNF/GO aerogel was immerged into 5 wt.% NaOH solution and equilibrated for 5 h. After desorption, the recovered aerogels were taken to separate from the solution and washed. Then, the final sample was freeze-dried under vacuum, followed by the next run of adsorption tests. The regenerated CNF/GO aerogel was reused for ten repeated cycles by following the above steps.

## Additional Information

**How to cite this article:** Yao, Q. *et al*. 3D assembly based on 2D structure of Cellulose Nanofibril/Graphene Oxide Hybrid Aerogel for Adsorptive Removal of Antibiotics in Water. *Sci. Rep.* **7**, 45914; doi: 10.1038/srep45914 (2017).

**Publisher's note:** Springer Nature remains neutral with regard to jurisdictional claims in published maps and institutional affiliations.

## Figures and Tables

**Table 1:** Kinetic properties pseudo-first-order and pseudo-second-order models for TC removal.

pseudo-first-order			pseudo-second-order		
Regression equation	k_1_	R^2^	Regression equation	k_2_	R^2^
In (*q*_1_ − *q*_t_) = 2.9379 − 0.7648*t*	0.76	0.83	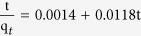	0.09	0.99

**Table 2:** Langmuir and Freundlich adsorption isotherms fitting parameters of TC.

Langmuir			Freundlich		
Regression equation	K_*L*_	R^2^	Regression equation	K_*F*_	R^2^
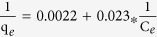	0.096	0.99	In*q*_*e*_ = 1.788 + 0.479InC_*e*_	61.4	0.98

**Table 3:** Maximum theoretical adsorption capacities (Q_*m*_) of antibiotics onto CNF/GO.

Antibiotic	Q_*m*_ (mg·g^−1^)	Antibiotic	Q_*m*_ (mg·g^−1^)
CAP	421.2	SQ	238.5
FF	418.7	SD	228.9
REM	307.5	ST	230.4
ETM	291.8	SM	327.8
NFC	134.6	SP	227.3
OFC	128.3	SMX	302.7
TAP	431.3	SMT	219.6
PNG	475.4	DXC	501.1
CFX	381.6	CTC	478.9
AXC	230.7	OTC	486.7
		TC	454.6

**Table 4:** 21 kinds of antibiotics and their abbreviations (Abb.).

Category	Name	Abb.	Category	Name	Abb.
Chloramphenicols	Chloramphenicol	CAP	Sulfonamides	Sulfaquinoxaline	SQ
	Florfenicol	FF		Sulfadiazine	SD
Macrolides	Roxithromycin	REM		Sulfamerazine	SM
	Erythromycin	ETM		Sulfapyridine	SP
Quinolones	Norfloxacin	NFC		Sulfamethazine	SMX
	Ofloxacin	OFC		Sulfamethoxazole	SMT
β-Lactams	Thiamphenicol	TAP	Tetracyclines	Doxycycline	CTC
	Peniciln G	PNG		Chlortetracycline	OTC
	Cefalexin	CFX		Oxytetracycline	TC
	Amoxicillin	AXC		Tetracycline	
